# Johann Gregor Mendel: paragon of experimental science

**DOI:** 10.1002/mgg3.199

**Published:** 2016-01-08

**Authors:** Mauricio De Castro

**Affiliations:** ^1^ United States Air Force Medical Genetics Center81st Medical GroupKeesler AFBBiloxiMS

## Abstract

This is a foreword on the life and work of one of the greatest minds of the 20th century, the father of modern genetics, Johann Gregor Mendel.

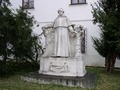

## Introduction

Much has been written about Gregor Mendel, posthumously recognized as the father of modern genetics. A man of many talents and inclinations, including Augustinian friar, botanist, horticulturist, meteorologist, apiculturist, viticulturist, astronomer, teacher, and mentor (Sorsby 1965; Richter 2015); clearly a multifaceted individual, a true renaissance man. Mendel's enduring legacy is describing the laws of inheritance and coining the terms dominant, recessive and discrete factor, a predecessor to the concept of gene (Orel 1984), all inferred from experiments carried out with his iconic peas (*Pisum sativum*).

It is hard to think of another human being that has created a more lasting impression in the field of Genetics than Gregor Mendel; the combination of the laws of inheritance with Darwin's theory of natural selection spawned the modern synthesis of evolutionary biology. As any clinical practitioner can attest to, it is impossible to escape Mendel's indelible impression in our field; we frequently talk about a trait or condition in terms of it being Mendelian (following his namesake laws of inheritance) or non‐Mendelian (mitochondrial inheritance for instance).

There is an extensive body of literature on all facets of the life of Gregor Mendel. Scholars of all walks of life have set out to explore and discuss the many different aspects of Mendel's work, which some readers may be surprised to know, were controversial for many years (Callender 1988; Fisher 1936; Weiling 1991; Brannigan 1979). This is by no means an exhaustive treatise on the life and work of the famous monk but meant instead, to act as a foreword. We have set out to lay bare the highlights of Mendel's life; to show how his industriousness and preparation in mathematics and physics laid the foundation for his analytical aptitude; how his meticulous data collection and inquisitive spirit were paramount in his studies and lastly, to illustrate how given the right circumstances, fate and external influences can sometimes conspire to create greatness.

It can be said that Mendel's achievements (like many other great scientists) are not exclusively his own, he had help along the way, sometimes from friends and family, sometimes from strangers, sometimes it seemed providence would conspire to help the young monk. We cannot help but think that Mendel would agree with this statement.

## Mendel's Humble Beginnings

**Figure 1:**
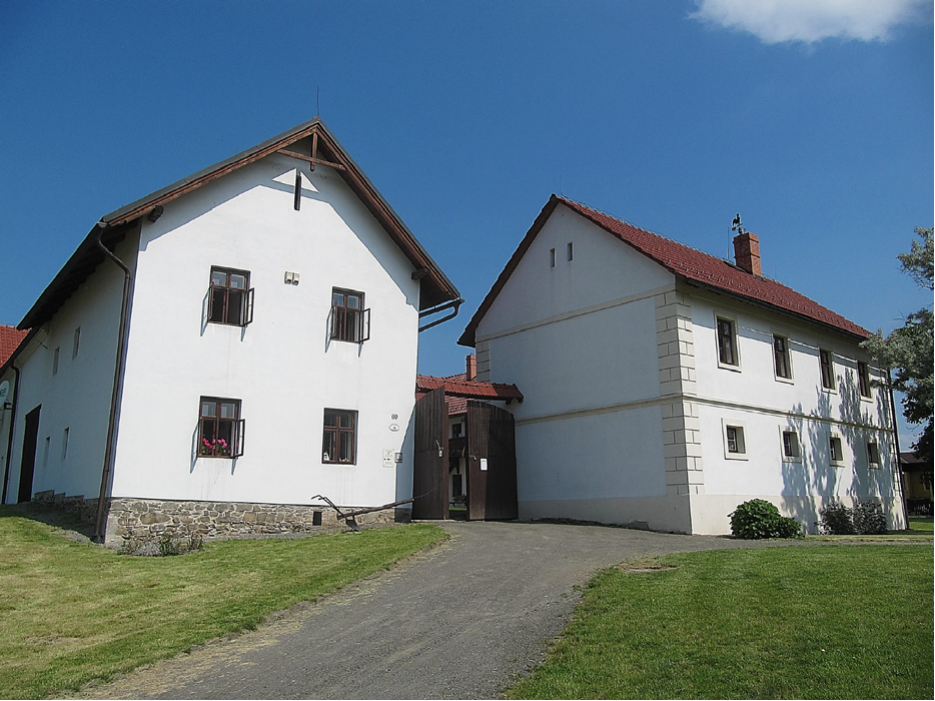
Birth house of Gregor Mendel in Heinzendorf bei Odrau. (Property of Palickap, Wikimedia commons).

Johann Mendel was born in Heinzendorf bei Odrau (Fig. 1), near the Moravian–Silesian border in what is now the Czech Republic (at that time part of the Austrian empire). The year was 1822, the day July 20th; born to a humble family of farmers in a predominantly German part of Northern Moravia, Mendel dutifully performed the duties of a farm boy until age 11. At that time, an impressed local schoolmaster, Johann Schreiber (himself, an accomplished horticulturist), recognizing his unusual intelligence and enthusiasm for learning, recommended the young Johann be sent to the gymnasium in Tropau (Opava). His family obliged despite the financial strain on them on account of his father's disability (victim of falling log accident).

Mendel graduated 6 years later in 1840 with honors, but his time in Tropau was not always auspicious, for 4 months he was incapacitated with what would become a frequent occurrence in Mendel's life (Dunn 2003). It has been noted by biographers that his father's accident and incapacitation likely had an impact on Mendel's own health; he was given to bouts of what could now be described as clinical depression.

After graduating from the gymnasium, Mendel went on to Olmutz and enrolled in the Philosophical Institute of the University of Olmutz (about 40 miles away) for a 2‐year program in practical and theoretical philosophy and physics; he did very well, especially in math and physics. Mendel found himself tutoring students as a side job to cover his expenses; his sister Theresia also helped pay for his studies with her dowry. Mendel finally graduated in 1843 (it took him longer than 2 years), after taking a hiatus during which he fought recurrent bouts of clinical depression. Mendel excelled in his final examination, especially in mathematics and physics; this would be a recurrent theme in Mendel's life. In Olmutz, Mendel met Johann Nestler, at the time, rector of the university and dean of natural history. Nestler, a notable biologist, was very interested in the rules of heredity and had carried out research dealing with animal and plant breeding; it is generally thought that he influenced young Johann at the time (Wood and Orel 2001).

## Mendel, Man of God and Science

**Figure 2:**
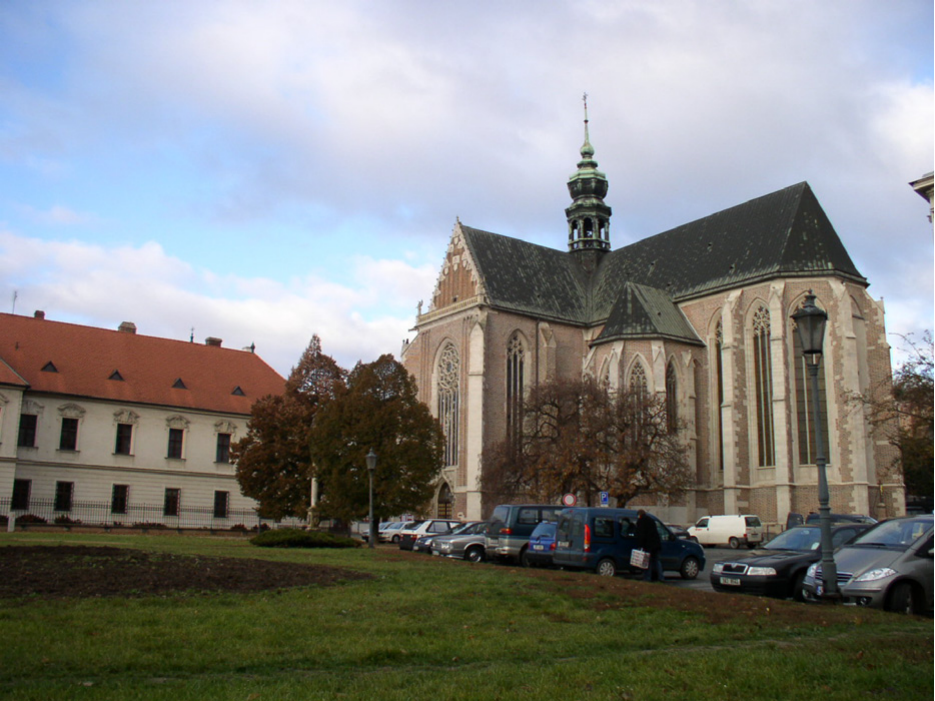
The Augustinian monastery where Mendel performed his experiments, in the southwest portion of old Brno. The church and the monk residency can be seen here. (Courtesy of Dr. David Fankhauser).

In 1844, at the age of 22, instead of going back to the family farm (as his father wanted) Johann decided to become a monk. To that end, he joined the Augustinian order at the St. Thomas Monastery in Brno (Fig. 2) (heeding the advice of his teacher at Olmutz, Friedrich Franz) and began his theological studies at the Episcopal seminary, taking the name Gregor. At the time, the monastery was the cultural center of the region; Mendel gained access to a huge library, and the research and teaching of various scientists. The monastery counted among its members well‐known philosophers, musicians, mathematicians, and botanists. At the time, the only route for higher education for the son of a farmer was through the church (Monaghan and Corcos 1993). The monastery's head, abbot Franz Cyril Napp who had more than a passing interest in the heredity traits of plants and animals, would play an important role in his life.

In 1846 Mendel took classes at the philosophical institute in Brno from Franz Diebl, an authority on plant breeding. He became ordained as a priest in 1847, after rapidly ascending through the steps of priesthood: novice, subdeacon, deacon and priest, getting his own parish in 1848 at the age of 26, the minimum at the time. The quickness through which he rose through the ranks was consequence of unfortunate circumstances: an infectious disease present in the town at the time had claimed the lives of three young priests just the year before in 1847 (Henig 2000).

Mendel's new clerical duties would prove to be too much for him; he fell ill once more around this time, unable to leave his bed. The monastery's head, abbot Napp thought that Mendel's skills would be put to better use elsewhere; in a letter to bishop Schaffgotsch in 1849 he remarked: “He is very diligent in the study of sciences but much less fitted for work as a parish priest, the reason being he is seized by an unconquerable timidity when he has to visit the ill. Indeed, this infirmity of his had made him dangerously ill”(Henig 2000); and so he was made a high school substitute teacher in 1849 in a secondary school in Znaim, where he would thrive. His colleagues, due to his “vivid and lucid method of teaching”, held Mendel in high esteem.

In 1850, at the age of 28, Mendel failed the final component of his teaching state certification examination, the oral portion. The following year, in 1851, Mendel was sent to Vienna at the behest of Abbot Napp and the suggestion of the state examiners to continue his studies in Science at the Royal Imperial University (Monaghan and Corcos 1993), the thought was that Mendel seemed to be incredibly bright but his lack of formal preparation put him at a disadvantage. In Vienna, he studied physics with Christian Doppler (of Doppler effect fame) and botany with Franz Unger. Unger had been using a microscope for his studies and was tinkering with a pre‐Darwinian theory of evolution, both are thought to have considerably shaped Mendel's scientific thought process and helped develop the skills he would put to good use later in his life, in particular the use of mathematics to evaluate and analyze empirical data (Henig 2000). Mendel's time in Vienna was the high point of his education; up to that time he had been largely self‐taught. Studying at the Imperial University marked the transition to educated man of science.

In 1853 after finishing his studies he came back to Brno and was given a position as a substitute schoolteacher in natural history and physics. In the spring of 1856 Mendel tried for the certification examination he had failed 6 years before; having spent time at Vienna and having practiced as a full time substitute teacher, Mendel thought himself prepared to retake the examination. Fortune would not smile on the monk that day; Mendel failed his certification examination once more, crippling testing anxiety and health issues being the likely culprits. Mendel would be relegated to being an uncertified, substitute teacher; only by virtue of being an excellent, enthusiastic educator was he able to retain his position; he would continue to teach in the lower two classes of secondary school. There was a silver lining to failing his certification examination; being a part‐time teacher allowed him to devote himself to his studies. From the year 1856 to 1863, Mendel diligently worked in his pea garden (Orel 1971).

## Mendel and his Peas

Blending was the prevailing theory at the time: the hereditary traits of offspring were the results of diluted blending of whatever traits were present in the parents. It was also commonly accepted that, over generations, a hybrid would revert to its original form. Farmers had known for millennia that selective breeding yielded favorable outcomes; Mendel was interested in better understanding how plant hybridization worked. The consensus appears to be that Mendel did not set out to prove the laws of inheritance (Opitz and Bianchi 2015; Olby 1979), instead, he worked with peas to develop new color variants and to examine the effects of hybridization. Mendel chose peas because they could be easily produced and cross‐fertilized and had many distinct characters (easy to phenotype).

**Figure 3:**
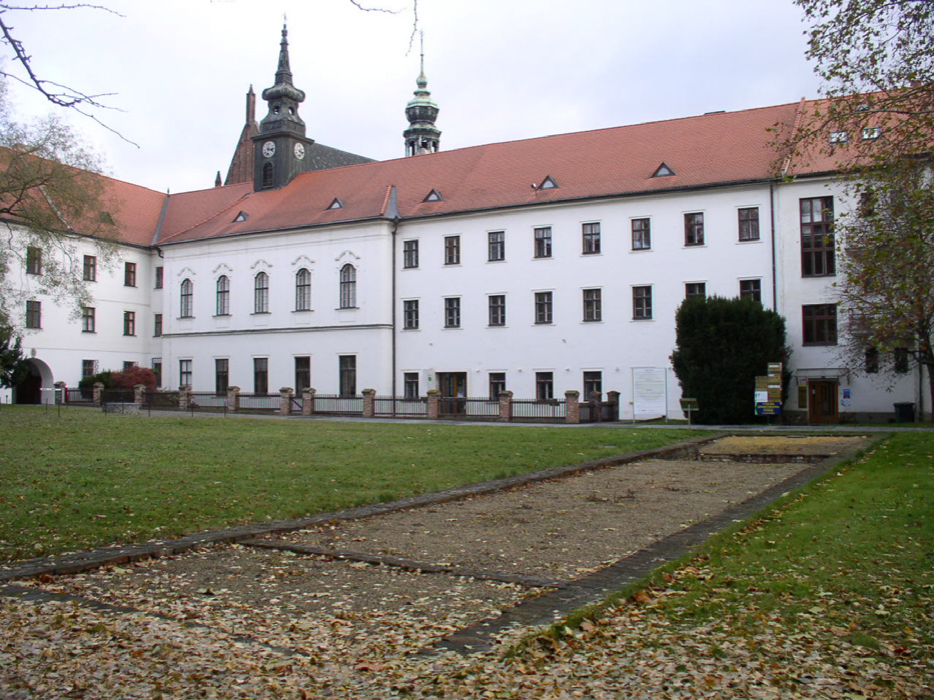
Grounds of the St. Thomas Abbey where Mendel bred his peas (*Pisum sativum*). The foundation of the greenhouse can still be seen in the proximal part of the picture. (Courtesy of Dr. David Fankhauser).

Mendel's selection of peas was quite serendipitous for reasons known and unknown to him. Peas exist in pure, separate lines; they are hermaphrodites and able to self fertilize before the bud opens (helps with contamination) and great numbers can be bred in a small space (Reid and Ross 2011). Unknown to Mendel is the fact that the characters he chose are not subject to linkage disequilibrium and most of them (at least 5 out of the 7 traits he used) are on separate chromosomes (Blixt 1975). This made the analysis of crosses and any conclusions inferred from them, straightforward. Mendel's pea experiments were carried out over 8 years and included more than 15,000 plants; astounding numbers, even by today's standards. The foundation of the greenhouse where he carried some of his experiments can still be seen today in St. Thomas monastery (Fig. 3).

Mendel's work with peas would be completed in 1863; the final analysis of the data and the preparation of the manuscript would happen in 1864. He finally submitted his seminal work on the laws of heredity, Versuche uber Pflanzen‐Hybriden (Experiments in plant hybridization) to the Proceedings of the Natural History Society of Brno in 1865, to be published in 1866. Mendel requested 40 copies of his paper; fourteen libraries in the United States currently have original copies of the 1866 Proceedings of the Natural History Society of Brno.

## Impact of Versuche

Mendel's paper had limited recognition upon its initial publication. It was mentioned in several publications over the next 34 years but its main thrust was never understood until later, 16 years after Mendel's death, in what is commonly referred to as the rediscovery (Orel 1971; Hartl and Orel 1992).

It was not until the 1900s when three botanists (Erich Tschermak in Austria, Hugo de Vries in the Netherlands, and Carl Correns in Germany) independently replicated his results. They found out after the fact, that the data and theory already had been published in 1866 by the Augustinian monk. Each scientist went to great lengths to show that they had read Mendel only after conducting their own experiments and reaching their own concussions (Weinstein 1977).

De Vries published first on the subject, mentioning Mendel in a footnote. Correns pointed out Mendel's priority after having read De Vries' paper and realizing that he himself did not have priority: “I thought that I had found something new. But then I convinced myself that the Abbot Gregor Mendel in Brno, had, during the sixties, not only obtained the same results through extensive experiments with peas, which lasted for many years, as did de Vries and I, but had also given exactly the same explanation, as far as that was possible in 1866” (Brannigan 1979).

De Vries may not have acknowledged truthfully how much of his knowledge of the laws came from his own work, or came only after reading Mendel's paper. It is speculated that De Vries had no intention of mentioning Mendel in his paper, only doing so after finding out that Correns and Tschermark had acknowledged Mendel's work (Sturtevant 1965).

There was also the issue of falsification of data. The now famous “goodness of fit” paper by Fisher suggested that some of Mendel's data was falsified; his conclusion based on statistical analysis of the data. Fisher had shown that the results obtained by Mendel were too close to what one would expect, suggesting that something other than chance was involved, there were too few random errors, his math was too precise; he quoted a one in 2000 chance that the experiments happened that way (Fisher 1936). It is important to point out that Fisher had much respect for Mendel and believed in his integrity despite the perception in some quarters that he exposed Mendel as a fraud (Edwards 1986). The criticism brought forth by Fisher found many advocates over the years and still to this day, some controversy remains amongst biologist and statisticians.

The publication of Mendel's paper would lay the foundation for what later became known as the particulate inheritance theory, articulated in later years by Bateson and Fisher. This theory would replace the blending model and pave the way for modern evolutionary synthesis and the birth of Genetics in the first part of the 20th century (Olby 1993).

## Mendel's Final Years

In 1868 Mendel replaced Napp as abbot of the monastery. From this point forward, his administrative duties took much of his time. In this stage of his life, Mendel would find himself isolated from his contemporaries by his public opposition to a new tax on monasteries in 1874. This battle continued until his death at the age of 62 in 1884, the official cause of death noted in the autopsy report is Bright disease (nephritis), with heart and kidney failure. His final years were consumed by his battle with the state over the taxes; overwhelmed by the administrative and clerical duties of his new position, he had to abandon his pea experiments.

**Figure 4:**
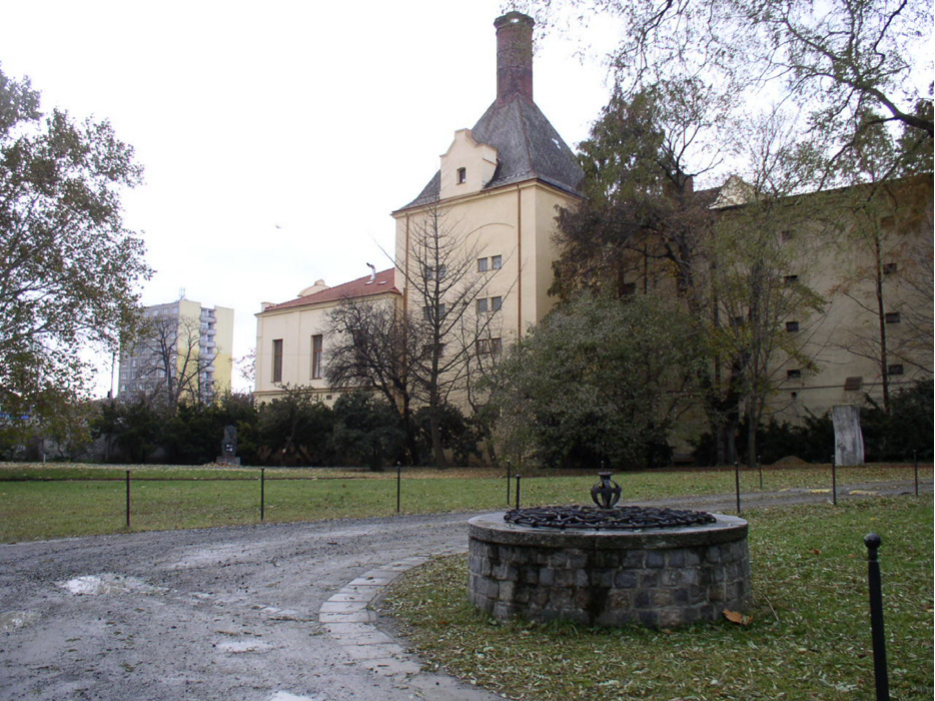
Mendel's well (Courtesy of Dr. David Fankhauser).

**Figure 5:**
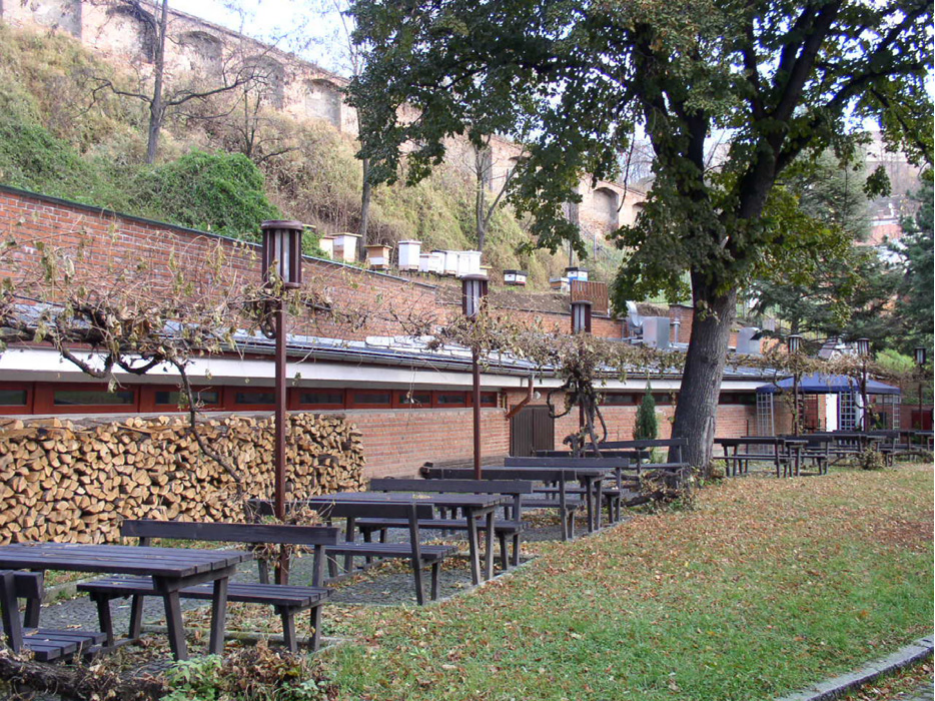
Mendel was an avid apiculturist. The abbey still keeps bees (Hives are visible on the terrace above the wall) (Courtesy of Dr. David Fankhauser).

**Figure 6:**
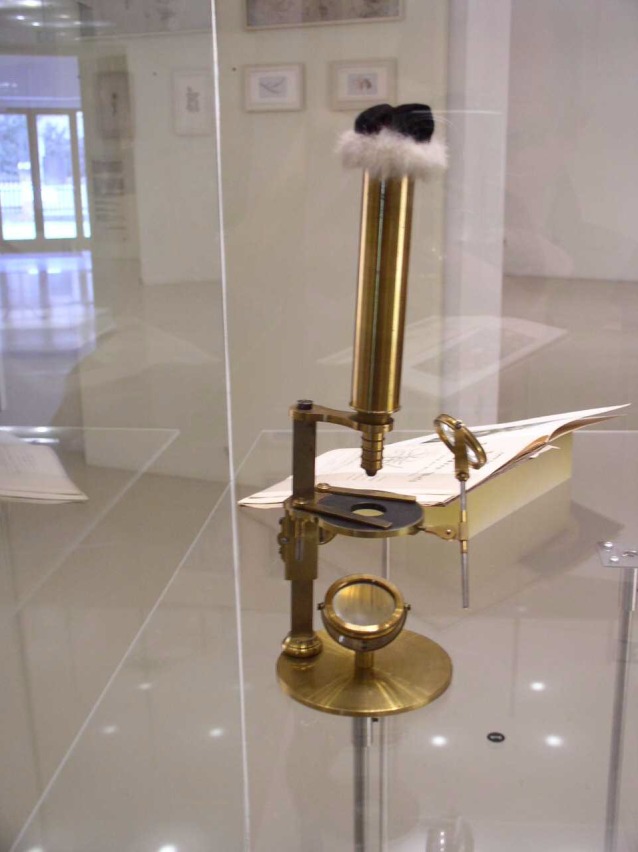
Mendel's microscope. (Courtesy of Dr. David Fankhauser).

Mendel pursued many other scientific interests throughout his life. In 1865, he founded the Austrian meteorological society (Mendel actually published more in meteorology than biology); he is known for careful, painstaking measurement of ground water (Fig. 4); hybridization experiments on other plants (*Hieracium*); vegetable and fruit tree horticulture (Walsh 1966); apiculture (Fig. 5 and Fig. 6) and agriculture in general (Weiling 1991). Mendel's data records on the water table, assessed from groundwater collection from a nearby well are one of the most impressive aspects of Mendel's extensive physical and meteorological observations. Conserved in the Mendel museum in Brno, are extensive records kept over the years of these data.

As it is often the case with historical figures, sometimes it is hard to accurately answer questions about their inner life, assess their personal motivations or intentions. In Mendel's case this is made the more difficult by the fact that many of his writings were destroyed in a fire to mark an end to disputes over taxation of religious institutions. Was Gregor Mendel a good natured monk who despite failing twice on his certification examinations, stumbled upon the laws of heredity by chance or even worse, falsified the data to make it look so? Did he really not understand the significance of his findings as some authors have suggested? (Monaghan and Corcos 1985), or was Mendel a quiet genius; an extraordinary monk who tirelessly worked to explain processes that had eluded the greatest minds of his time, to include Charles Darwin. Through much of the 20th century a debate raged on the meaning of Mendel's experiments. Although there was widespread agreement on the importance of his work to modern biology, there was much questioning on his protocols, his motives, and his own beliefs about evolution and heredity (Singh 2015).

Mendel had a difficult life, filled with obstacles and disappointments but also with many happy times and remarkable successes. His father's accident and the family's financial limitations had a deep impact on Mendel. Failing twice on the state certification examinations also affected him considerably. In his final years, the protracted fight with the government over the taxing of religious institutions finally took its toll on Mendel. These obstacles have to be contrasted with all the happy hours spent doing what he did best, the pursue of science; with his bees and his garden, collecting groundwater data, making meteorological observations, and of course his greatest success, elucidating the laws of heredity. Mendel's training in physics and mathematics (Teicher 2014), his meticulous data collection, his extraordinary attention to detail, gave him an advantage; he had the appropriate background for it. In many ways, he is the poster boy model for experimental science, carrying out his experiments over 8 years, overseeing more than 25,000 plants, and then, assiduously collecting, compiling, analyzing the data, and formulating hypotheses based on mathematical models.

**Figure 7:**
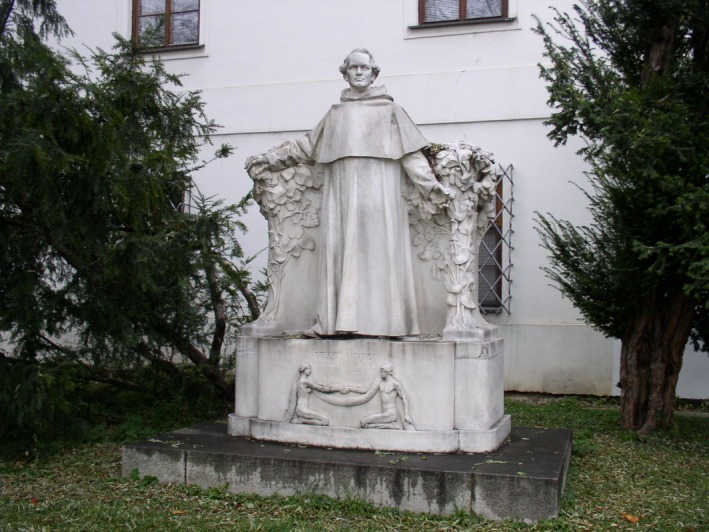
Mendel's memorial statue watching over his garden in the old Brno monastery. (Courtesy of Dr. David Fankhauser).

Even though it took decades for Mendel to take his rightful place in the pantheon of science's greatest minds, I would like to think that he would be very happy with the direct and indirect consequences of his studies; he would be honored to know that the Genetics community regards him as a founding father. In closing, I think I will let Mendel himself have the last thought: “I have experienced many a bitter hour in my life. Nevertheless, I admit gratefully that the beautiful, good hours far outnumbered the others. My scientific work brought me such satisfaction, and I am convinced the entire World will recognize the results of these studies”. Mendel was right, recognized we have (Fig. 7).

## Conflict of Interest

None declared.
